# Exposure to cyclooxygenase-2 inhibitors and risk of cancer: nested case–control studies

**DOI:** 10.1038/bjc.2011.252

**Published:** 2011-07-12

**Authors:** Y Vinogradova, C Coupland, J Hippisley-Cox

**Affiliations:** 1 Division of Primary Care, 13th Floor, Tower Building, University Park, University of Nottingham, Nottingham, NG7 2RD, UK

**Keywords:** COX2 inhibitors, breast cancer, colorectal cancer, haematological cancers, QResearch

## Abstract

**Background:**

Selective cyclooxygenase-2 (COX2) inhibitors are widely used as analgesics and it is unclear whether its long-term use affects cancer risk.

**Methods:**

A series of nested case–control studies using the QResearch primary care database. Associations of COX2 inhibitor use with risk of all cancers and 10 common site-specific cancers were estimated using conditional logistic regression adjusted for comorbidities, smoking status, socioeconomic status, and use of non-steroidal anti-inflammatory drugs, aspirin and statins.

**Results:**

A total of 88 125 cancers, diagnosed between 1998 and 2008, matched with up to five controls, were analysed. Use of COX2 inhibitors for more than a year was associated with a significantly increased risk of breast cancer (odds ratio (OR) 1.24, 95% confidence interval (CI) 1.08–1.42) and haematological malignancies (OR 1.38, 95% CI 1.12–1.69) and a decreased risk of colorectal cancer (OR 0.76, 95% CI 0.63–0.92). There were no other significant associations.

**Conclusion:**

Prolonged use of COX2 inhibitors was associated with an increased risk of breast and haematological cancers and decreased risk of colorectal cancer. These findings need to be confirmed using other data sources.

Selective cyclooxygenase-2 (COX2) inhibitors are used for patients intolerant to traditional non-steroidal anti-inflammatory drugs (NSAID), which have gastrointestinal toxic effects (Chan *et al*, 2010). Being introduced in the United Kingdom in 1985, COX2 inhibitors account for 14% of all NSAIDs prescriptions (The NHS Information Centre for health and social care, 2008), despite advice from the UK Medicines and Healthcare products Regulatory Agency (MHRA, 2005) about possible cardiovascular adverse effects (Solomon *et al*, 2005).

Laboratory investigations have suggested mechanisms by which COX2 inhibitors might reduce the risk of cancer (Koki and Masferrer, 2002; Khan and Lee, 2009) for a range of cancers, although animal experiments have not provided consistent support. A recent publication, for example, shows that COX2 inhibitors do not delay or prevent tumour development in breast tissue in a mouse model (Tran-Thanh *et al*, 2010).

Some observational studies have investigated effects of COX2 inhibitors on cancer risk, but have produced inconsistent results (Arber *et al*, 2006; Harris *et al*, 2006, 2007; Hernández-Díaz and García Rodríguez, 2006). For colorectal cancer, a randomised control trial (Arber *et al*, 2006) showed a 36% decreased rate of newly detected colorectal adenomas in celecoxib users. Two studies (Harris *et al*, 2006, 2007) demonstrated risk reductions for breast and lung cancer, but a larger case–control study (Hernández-Díaz and García Rodríguez, 2006) using primary care data showed no effect for lung cancer. Effects on other cancers remain unclear.

We designed a series of large-scale nested case–control studies to determine associations between selective COX2 inhibitors and risks of common cancers. We used the QResearch primary care database, which is large, has a representative population and contains data for individual drug exposures and outcomes.

## Materials and methods

### Study design, data source and population

We conducted a series of nested case–control studies using version 20 of the QResearch primary care database (http://www.qresearch.org) containing anonimised clinical records for over 11 million patients registered with 574 UK general practices. The information recorded on the database includes patient demographics (year of birth, sex, sociodemographic data derived from the UK census 2001), characteristics (height, weight, smoking status), clinical diagnoses, symptoms, consultations, referrals, prescribed medications and results of investigations. The database has been validated by comparing birth rates, death rates, consultation rates, prevalence and mortality rates with other data sources, including the General Household Survey and the General Practice Research Database (National Statistics, 2000; Hippisley-Cox *et al*, 2005).

We initially identified an open cohort of patients registered between 1 Jan 1997 and 1 July 2008 with participating UK general practices. We then selected as cases all those patients in the cohort aged between 30 and 100 years with a first-ever recorded diagnosis of cancer during the study period, identified from diagnostic READ codes in patient records (the standard clinical terminology system used in General Practice in the UK (Smith *et al*, 1995)). Each case was linked to five controls who were alive, had no history of cancer and were registered with the practice at the time of case diagnosis (the index date), matched on age, sex, practice and calendar time using incidence-density sampling.

### Exclusions

Cases with secondary cancers (READ codes: B56, B57, B58) and non-melanoma skin cancer were excluded. For breast cancer, we included only females, and excluded cases and controls with a record of mastectomy or tamoxifen use for more than 12 months before the index date to exclude possible previous diagnoses. We also excluded temporary residents and patients with fewer than 6 years of medical records before the index date to ensure completeness of exposure data.

### Primary outcomes

We analysed cancers overall, and carried out separate analyses for the most common UK cancers (Westlake, 2008): breast (women, B34), prostate (men, B46), lung (B22), colorectal (B13, B14), haematological (B6), bladder (B49), melanoma (B32), gastric (B11), pancreatic (B17) and oesophageal (B10). As haematological malignancies cover a range of diseases, possibly differentially affected by COX2 inhibitors (Nakanishi *et al*, 2001; Nakamura *et al*, 2006), we also investigated leukaemia (B63-B6z), lymphoma (B60-B62) and myeloma (B63) separately.

### Data

Records in the year before the index date were ignored to reduce protopathic bias. Prescriptions for cases in this period could relate to early cancer symptoms before the recorded diagnosis. All analyses were, therefore, based only on prescriptions relating to the period between 13 and 72 months before the index date.

We assessed exposure to COX2 inhibitors, including celecoxib, etodolac, etoricoxib, lumiracoxib, rofecoxib, valdecoxib, meloxicam (British Medical Association, Royal Pharmaceutical Society, 2010). We also extracted data on prescriptions for statins, traditional NSAIDs and aspirin because studies have found protective effects of these on various types of cancer (Garcia Rodriguez and Huerto-Alvarez, 2001; Sørensen *et al*, 2003; Jacobs *et al*, 2005; Bardia *et al*, 2007; Gallicchio *et al*, 2007), in particular, colorectal cancer (Garcia Rodriguez and Huerto-Alvarez, 2001; Sørensen *et al*, 2003).

We extracted information on age and sex, smoking status (non-smoker, ex-smoker, current smoker), body mass index (BMI) in kg m^−2^, Townsend score (measure of socioeconomic status) and data on comorbidities (cardiovascular disease, hypertension, diabetes, rheumatoid arthritis and osteoarthritis). For breast cancer, we also accounted for previous benign breast disease (fibrocystic disease, intraductal papilloma, fibroadenoma), family history of breast cancer, use of hormone replacement therapy and oral contraceptives. For colorectal cancer, additional comorbidities were ulcerative colitis and Crohn's disease.

We considered patients as COX2 inhibitor users if they had at least one prescription. We estimated cumulative use of COX2 inhibitors by extracting the duration of use for every prescription and, for groups of prescriptions with inter-prescription gaps of less than 60 days; we calculated overall course times from the start of the first prescription to the end of the last prescription. We then calculated cumulative use as the sum of all overall course times and categorised cumulative use for each patient as: no use, less than 90 days, 90 days to 12 months; 13–24 months; 25–60 months. We also categorised cumulative use as: no use; short-term use (less than 365 days) and long-term use (more than 365 days). A trend test was performed using the actual months of use. We conducted separate analyses for the most common individual COX2 inhibitors – meloxicam, rofecoxib and celecoxib, examining the effect on cancer risk of cumulative use for more than 365 days.

The daily dose of COX2 inhibitors was estimated as the median daily dose of all prescriptions of any COX2 drug recorded. It was categorised by COX2 inhibitor efficacy (Hernández-Díaz and García Rodríguez, 2006) as: high (for celecoxib >200 mg, for meloxicam >7.5 mg, for rofecoxib >25 mg, for etodolac >400 mg, for etoricoxib >90 mg, for valdecoxib >40 mg, for luminoracoxib >200 mg); otherwise as low/medium.

The effect on cancer risk of stopping COX2 inhibitors for long-term and short-term users was investigated by determining the last prescription date and categorising each patient at 12 months before the index date as: no COX2 inhibitors use, current COX2 inhibitors user, recent user (stopped the drugs at 13–24 months before the index date) and past user (stopped the drugs at 25 or more months before the index date).

### Statistical analysis

We used conditional multivariate logistic regression to estimate odds ratios (ORs) with 95% confidence intervals (CIs) associated with COX2 inhibitor use compared with non-use for cancers overall and each specific cancer. We calculated unadjusted ORs and adjusted for the potential confounding variables listed above, in which patients were classified as users of each medication if they had at least one prescription for NSAIDs or aspirin and at least two prescriptions for statins, hormone replacement therapy and oral contraceptives.

We carried out multiple imputation (Royston, 2005) with Stata ICE programs to replace missing values of BMI, smoking status and Townsend deprivation scores. We applied Rubin's rules to five imputed data sets to combine effect estimates for each cancer separately. We removed rheumatoid arthritis patients in an additional analysis to eliminate its potential effect on the risk of haematological malignancies Thomas *et al*, 2000).

We used all the available data on the QResearch database, hence, did not do a pre-study sample size calculation. We chose a 1% significance level to determine statistical significance to account for the multiple outcomes. Stata v10 (StataCorp LP, College Station, TX, USA) was used for all analyses.

## Results

There were 118 780 patients with diagnoses of cancers in the study period matched with 588 797 controls. Of the patients with cancer, 3810 with secondary cancers and 36 with inapplicable cancers (e.g., male/cervical cancer) were removed. For breast cancer, 1055 cases and 773 controls with a previous mastectomy or tamoxifen use were excluded. This left 113 879 cases with a first diagnosis of cancer during the study period and 568 958 matched controls. After removing 25 754 cases and 206 704 controls with <6 years of medical records or lacking a matched case or control, there were 88 125 cases of primary cancer matched with 362 254 controls, which were used in the analyses. The proportions of each cancer type in cases matched registration statistics in England for 2007 (Statistical Bulletin, 2010) for patients older than 30 years.

### Baseline characteristics

Table 1 shows baseline characteristics for cases and controls. Fifty-three percent of cases were men; with a median age at diagnosis of 69 years (interquartile range: 60–77). Overall, 76% of cases and 73% of controls had complete data for BMI, smoking status and Townsend deprivation score. Cases and controls had similar patterns of comorbidity.

### Exposure to COX2 inhibitors

**Figure 1:**
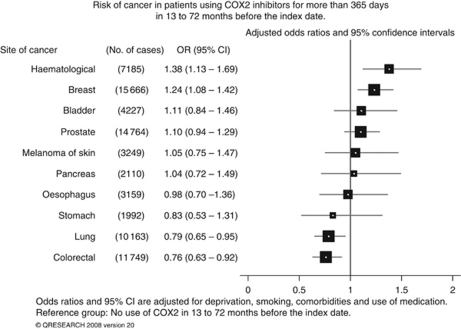
Risk of cancer in patients using COX2 inhibitors for more than 365 days in 13–72 months before the index date.

Overall 7.8% (6901) of cases and 7.4% (26 974) of controls had at least one prescription for COX2 inhibitors. Most users (70% cases, 70% controls) had no gap longer than 60 days between the first and last prescription, with 19% cases and 19% controls having only one gap longer than 60 days. Twenty-one percent of COX2 inhibitor users (21% cases, 21% controls) had prescriptions for more than 365 days (Figure 1) with median 20 prescriptions (interquartile range, 14–30 for cases, 14–31 for controls). Median duration of use for these long-term users was 25 months (interquartile range 17–37 for cases and 18–37 for controls) and median duration for short-term users was 2 months (interquartile range 1–4 for both cases and controls).

The most frequently prescribed COX2 inhibitors were rofecoxib (3.1% cases, 3.0% controls), celecoxib (2.6% cases, 2.5% controls) and meloxicam (2.6% cases, 2.4% controls). Other COX2 inhibitors were prescribed to <1% cases and 1% controls. Most rofecoxib users were on low/medium dose (71% cases, 72% controls), most celecoxib users were on high dose (81% cases, 79% controls) and more than half of meloxicam users (65% cases, 65% controls) were on high dose.

A higher proportion of COX2 inhibitors users had hypertension, cardiovascular disease, diabetes, rheumatoid arthritis and osteoarthritis than non-users (Table 2).

#### Cancer of any site

The analysis for cancer risk showed a significant association with any COX2 inhibitors use, although the OR (Table 3) was close to unity (OR 1.06, 95% CI 1.03–1.09, *P*<0.001), and no association for long-term use (OR 1.02, 95% CI 0.96–1.08, *P*=0.616). Analyses of trends for duration of use and dosage, as well as individual COX2 inhibitors use did not show significant associations with overall cancer risk (Table 4 and Supplementary information).

#### Colorectal cancer

There was no association between any use of COX2 inhibitors and risk of colorectal cancer, but the association with long-term use was significant (OR 0.76, 95% CI 0.63–0.92, *P*=0.004). There was a significant trend for duration of use (*P*_trend_=0.004) with an OR of 0.66 (95% CI 0.51–0.86, *P*=0.002) for more than 24 months of use. Risk of colorectal cancer stayed significantly decreased for long-term users who stopped COX2 inhibitors more than 2 years before the index date (OR 0.74, 95% CI 0.60–0.92, *P*=0.007).

#### Breast cancer

Risk of breast cancer was not statistically significantly associated with overall COX2 inhibitor use, but there was a significant trend with duration of use (*P*_trend_=0.002) with an increased risk in long-term users (OR 1.24, 95% CI 1.08–1.42, *P*=0.003), which stayed increased after stopping COX2 inhibitors more than 2 years before the index date (OR 1.23, 95% CI 1.05–1.44, *P*=0.009).

#### Haematological malignancies

There was a significant association between risk of haematological malignancies and COX2 inhibitor use (OR 1.18, 95% CI 1.07–1.31, *P*=0.001) with an even stronger association for long-term users (OR 1.38, 95% CI 1.12–1.69, *P*=0.002). There was a significant trend for duration of use (*P*_trend_< 0.001) and an increased risk of 47% in users for more than 2 years (*P*=0.008). Removing cases and controls with rheumatoid arthritis did not change the ORs. Meloxicam had the highest OR (OR 1.27, 95% CI 1.08–1.50, *P*=0.004) for overall use, but others were not statistically significant. The risk in long-term users remained significantly increased after stopping COX2 inhibitors for more than 2 years before the index date (OR 1.40, 95% CI 1.11–1.76, *P*=0.005).

The ORs for overall use in separate analyses for leukaemia, lymphoma and myeloma showed consistent increases, though only myeloma was significant (ORs 1.18, 95% CI 1.02–1.36, *P*=0.030; 1.21, 95% CI 1.01–1.45, *P*=0.036; and 1.43, 95%CI 1.13–1.81. *P*=0.003, respectively). Long-term use showed a stronger effect for lymphoma (ORs 1.20, 95% CI 0.88–1.64, *P*=0.246; 1.70, 95% CI 1.21–2.40, *P*=0.002; and 1.38, 95% CI 0.87–2.19, *P*=0.168, for leukaemia, lymphoma and myeloma, respectively), with respective trends (*P*_trend_=0.071), (*P*_trend_=0.001) and (*P*_trend_=0.048) for actual months of use.

#### Lung cancer

There were no significant associations for lung cancer. Long-term COX2 inhibitor users had a lower risk (OR 0.79, 95% CI 0.65–0.95, *P*=0.012), but it was not statistically significant at the level of 0.01.

#### Other cancers

There were no significant associations with COX2 inhibitor use for other cancers.

#### Other analyses

No dose–response association with cancer was found for any site. No particular type of COX2 inhibitor overall use was associated with increased or decreased risk of cancer (except for blood cancer reported above).

## Discussion

The key findings from our study are that long-term use of selective COX2 inhibitors was associated with a 24% reduced risk of colorectal cancer, a 24% increased risk of breast cancer and a 38% increased risk of haematological cancer. No significant increases or decreases for other common cancers were found. Although the protective effect for colorectal cancer might have been hypothesised from theoretical and laboratory studies (Koki and Masferrer, 2002; Khan and Lee, 2009), we believe this is the first demonstration using general population clinical data.

### Comparison with other studies

Many epidemiological studies have investigated the effects of nonspecified or combined (COX2 and traditional) NSAIDs on cancer risk (Garcia Rodriguez and Huerto-Alvarez, 2001; Sørensen *et al*, 2003; Jacobs *et al*, 2005; Hernández-Díaz and García Rodríguez, 2006; Bardia *et al*, 2007; Gallicchio *et al*, 2007). A number of them have suggested overall chemoprotective properties of NSAIDs for several cancers, in particular colorectal (Garcia Rodriguez and Huerto-Alvarez, 2001; Sørensen *et al*, 2003) and, for long-duration regular users, lung, prostate and breast cancer (Jacobs *et al*, 2005; Hernández-Díaz and García Rodríguez, 2006; Gallicchio *et al*, 2007).

There is less evidence for newer COX2 drugs, although laboratory and animal studies (Liu *et al*, 2004; Manish *et al*, 2005; Barnes *et al*, 2007; D’Arca *et al*, 2010) using COX2 inhibitors have shown possible decreases in cancer incidence. The reduced risk of colorectal cancer in our study was comparable with the 56% decreased risk of distal large bowel cancer in COX2 inhibitor users (Kim *et al*, 2008). COX2 inhibitor chemoprotective effects were also demonstrated in a randomised controlled trial for colorectal cancer prevention (Arber *et al*, 2006), although on patients with increased baseline risk because of previous history of adenomas. Although the trial was planned for 5 years of surveillance and treatment, it was stopped after 3.1 years because of adverse cardiovascular effects, but it still demonstrated a significant anti-tumour effect with risk reductions of 55–67% depending on celecoxib dose (Bertagnolli *et al*, 2006).

Our study's finding of an increased risk of breast cancer contrasts with findings from a hospital-based case–control study on selective COX-2 inhibitors (Harris *et al*, 2006), which demonstrated a significant risk reduction (OR 0.29, 95% CI 0.14–0.59) with daily use for at least 2 years. This study was very small (only 10 cases), and used questionnaire data and hence would have been subject to recall bias. Another study (Rahme *et al*, 2005) on menopausal women showed a reduction in breast cancer risk (OR 0.81, 95% CI 0.68–0.97) for COX2 inhibitor use of 90 days or longer, however, with shorter exposure (average of eight prescriptions). Although no other recent epidemiological study has looked at specific effects of COX-2 inhibitors, a number of studies have investigated effects of nonspecified or combined NSAID use on breast cancer (Gill *et al*, 2007; Kirsh *et al*, 2007; Ready *et al*, 2008), mostly finding no association. The mechanism of inhibiting of COX2 expression might differ for different types of traditional NSAIDs, and a cohort study (Marshall *et al*, 2005) demonstrated an increased risk in ibuprofen users but not in aspirin or other NSAIDs.

We showed increased risks for haematological malignancies, particularly lymphoma. Frequent traditional NSAID users with rheumatoid arthritis may have double the risk of having haematological cancers (Thomas *et al*, 2000), and one rheumatoid arthritis study showed an increased risk of lymphoma (Baecklund *et al*, 2006) from chronic inflammation. Removing rheumatoid arthritis patients, left our results unchanged, suggesting an effect from COX2 inhibitors rather than from the condition. Another meta-analysis demonstrated no association between NSAIDs and non-Hodgkin lymphoma (Bernatsky *et al*, 2007) risk, but the only study on COX2 inhibitors found a possible increased risk associated with regular use (Flick *et al*, 2006) (OR 1.58, 95% CI 0.68–3.67). A recent study (Chang *et al*, 2010) also demonstrated an increased risk of Hodgkin lymphoma, associated with COX2 inhibitors.

There is no established biological mechanism explaining the associations between COX2 inhibitors and risk of breast or blood cancers, and further exploration is needed.

We found no significant reduction of lung cancer risk in patients with over 1 year use of COX2 inhibitors, although there was some indication of a decreased risk (OR 0.79, 95% CI 0.65–0.95), in contrast to a very small study reporting a 60% reduction for COX2 inhibitor use of 2 years or more (Harris *et al*, 2007) (22 cases) with inevitable recall bias. A larger case–control study demonstrated a reduction of risk (Hernández-Díaz and García Rodríguez, 2006), based on all NSAIDS, but no significant association for COX2 inhibitors.

### Strengths and limitations

The study was substantially larger than earlier studies, including information from all patients, including those with short survival. There is no recall bias, as details of prescriptions and confounding factors were recorded prospectively before the index date. Bias from misclassification of diagnoses was unlikely because accuracy and completeness of records in general practices is high (Hippisley-Cox *et al*, 2003; Herrett *et al*, 2010). Matching controls on sex, age, practice and calendar year removed effects from these confounding factors and we adjusted for a number of other confounding variables. Although we used a 1% level to define statistical significance level, some of our findings might still have arisen from multiple significance testing. Bias from misclassification of COX2 inhibitor use was unlikely as over 99% of all repeat prescriptions are computer recorded (Department of Health, 2007), and underestimation of use was unlikely as these drugs are prescription-only.

We did not adjust for certain cancer risk factors, such as physical activity, women's reproductive history, alcohol use and diet, because these are not consistently recorded. There may, therefore, be residual confounding if these factors are associated with COX2 inhibitor use. Body mass index, smoking status and deprivation had missing values in 22% of cases and in 25% of controls, and we used multiple imputation to replace these values. Although our data contain detailed information on drug prescriptions, this may not reflect the actual use. There is no reason to think that any non-adherence would systematically differ between cases and controls, however, such misclassification might have biased the ORs towards one making the associations weaker. There may be residual confounding because of over-the-counter use of NSAIDs and aspirin, which was not accounted for in the analyses. There was no information about cancer stage and it is unknown whether the symptoms before diagnosis led to COX2 inhibitor use. The possibility of this was minimised by ignoring prescriptions in the last year before the index date.

### Summary

We have conducted a large population-based case–control study examining the association of selective COX-2 inhibitors with risk of common cancers in the general population and found a reduced risk of colorectal cancer, but increased risks of breast and haematological malignancies in long-term COX2 inhibitor users, which did not decrease after cessation. This was a very broad study covering a range of cancers, each of which, though related, are complex and exhibit significant variations in terms of disease mechanisms and progression, symptoms and treatments. The primary value of the study is, therefore, as a comprehensive overview, identifying the relative potential of different areas for further focused investigation. Although some significant findings are reported, further studies are suggested, in particular, in the areas of breast and blood cancers.

## Figures and Tables

**Table 1:** Baseline characteristics for all cases with primary cancer and their matched controls with at least 6 years of medical records

	**Cases (*N*=88 125)**	**Controls (*N*=362 254)**
*Sex*		
Female	41 749 (47.4)	170 173 (47.0)
Male	46 376 (52.6)	192 081 (53.0)
		
*Age band (years)*		
30–54	13 151 (14.9)	49 906 (13.8)
55–64	19 638 (22.3)	80 107 (22.1)
65–74	26 758 (30.4)	111 698 (30.8)
75–84	25 013 (28.4)	106 278 (29.3)
85+	3565 (4.0)	14 265 (3.9)
		
*Deprivation, Townsend quintile*		
1, Most affluent	22 072 (25.0)	92 287 (25.5)
2	18 998 (21.6)	79 067 (21.8)
3	17 338 (19.7)	71 358 (19.7)
4	15 325 (17.4)	61 767 (17.1)
5, Most deprived	11 896 (13.5)	45 971 (12.7)
Townsend missing	2496 (2.8)	11 804 (3.3)
		
*Body mass index (kg m* ^*−2*^ *)*		
15–24	26721 (30.3)	105 883 (29.2)
25–29	27 285 (31.0)	108 803 (30.0)
30–49	12 922 (14.7)	51 413 (14.2)
Not recorded	21 197 (24.1)	96 155 (26.5)
		
*Smoking status*		
Non-smoker	54 307 (61.6)	233 135 (64.4)
Ex-smoker	7567 (8.6)	23 842 (6.6)
Current smoker	17 275 (19.6)	54 869 (15.1)
Not recorded	8976 (10.2)	50 408 (13.9)
		
*Comorbidities*		
Cardiovascular disease	14 278 (16.2)	58 123 (16.0)
Diabetes	7115 (8.1)	26 802 (7.4)
Hypertension	27 104 (30.8)	109 797 (30.3)
Osteoarthritis	12 807 (14.5)	52 586 (14.5)
Rheumatoid arthritis	1310 (1.5)	5132 (1.4)
Colitis^a^	124 (1.1)	293 (0.6)
Crohn's disease^a^	28 (0.2)	109 (0.2)
Benign breast disease^b^	1094 (7.0)	2937 (4.7)
Family history of breast cancer^b^	539 (3.4)	1249 (2.0)
		
*Medications (in previous 13–72 months)*		
Traditional NSAIDs	35 697 (40.5)	140 642 (38.8)
Aspirin	19 895 (22.6)	79 067 (21.8)
Statins	13 621 (15.5)	54 606 (15.1)
Hormone replacement therapy^b^	3289 (21.0)	10 973 (17.4)
Oral contraceptive pill^b^	523 (3.3)	1638 (2.6)

Abbreviation: NSAID=non-steroidal anti-inflammatory drug.

a On the basis of cases with colorectal cancer and their controls only.

b On the basis of female cases with breast cancer and their controls only.

Values are shown as numbers and %.

**Table 2:** Baseline characteristics in cases and controls COX2 users and non-users (at least one prescription in 13 to 72 months before index date)

	**Cases (*N*=88 125)**		**Controls (*N*=362 254)**	
	**COX2 user**	**COX2 non-users**	**COX2 user**	**COX2 non-users**
	***N*=6901**	***N*=81 224**	***N*=26 974**	***N*=335 280**
*Sex*				
Female	3833 (55.5)	37 916 (46.7)	15 055 (55.8)	155 118 (46.3)
Male	3068 (44.5)	43 308 (53.3)	11 919 (44.2)	180 162 (53.7)
				
*Age band (years)*				
30–54	455 (6.6)	12 696 (15.6)	1693 (6.3)	48 213 (14.4)
55–64	1309 (19.0)	18 329 (22.6)	4830 (17.9)	75 277 (22.5)
65–74	2123 (30.8)	24 635 (30.3)	8616 (31.9)	103 082 (30.7)
75–84	2466 (35.7)	22 547 (27.8)	9911 (36.7)	96 367 (28.7)
85+	548 (7.9)	3017 (3.7)	1924 (7.1)	12 341 (3.7)
				
*Deprivation, Townsend quintile*				
1, Most affluent	1606 (23.3)	20 466 (25.2)	6378 (23.6)	85 909 (25.6)
2	1467 (21.3)	17 531 (21.6)	5876 (21.8)	73 191 (21.8)
3	1344 (19.5)	15 994 (19.7)	5491 (20.4)	65 867 (19.6)
4	1334 (19.3)	13 991 (17.2)	4933 (18.3)	56 834 (17.0)
5, Most deprived	955 (13.8)	10 941 (13.5)	36 39 (13.5)	42 332 (12.6)
Townsend missing	195 (2.8)	2301 (2.8)	657 (2.4)	11 147 (3.3)
				
*Body mass index (kg m* ^*−2*^ *)*				
15–24	1802 (26.1)	24 919 (30.7)	7228 (26.8)	98 655 (29.4)
25–29	2491 (36.1)	24 794 (30.5)	9686 (35.9)	99 117 (29.6)
30–49	1482 (21.5)	11 440 (14.1)	5814 (21.6)	45 599 (13.6)
Not recorded	1126 (16.3)	20 071 (24.7)	4246 (15.7)	91 909 (27.4)
				
*Smoking status*				
Non-smoker	4659 (67.5)	49 648 (61.1)	19 789 (73.4)	213 346 (63.6)
Ex-smoker	742 (10.8)	6825 (8.4)	2346 (8.7)	21 496 (6.4)
Current smoker	1215 (17.6)	16 060 (19.8)	3661 (13.6)	51 208 (15.3)
Not recorded	285 (4.1)	8691 (10.7)	1178 (4.4)	49 230 (14.7)
				
*Comorbidities*				
Cardiovascular disease	1483 (21.5)	12 795 (15.8)	5991 (22.2)	52 132 (15.5)
Diabetes	711 (10.3)	6404 (7.9)	2601 (9.6)	24 201 (7.2)
Hypertension	2858 (41.4)	24 246 (29.9)	11 449 (42.4)	98 348 (29.3)
Osteoarthritis	2667 (38.6)	10 140 (12.5)	10 623 (39.4)	41 963 (12.5)
Rheumatoid arthritis	400 (5.8)	910 (1.1)	1519 (5.6)	3613 (1.1)
Colitis^a^	8 (0.9)	116 (1.1)	34 (0.9)	259 (0.6)
Crohn's disease^a^	2 (0.2)	26 (0.2)	9 (0.2)	100 (0.2)
Benign breast disease^b^	92 (7.1)	1002 (7.0)	210 (4.2)	2727 (4.7)
Family history of breast cancer^b^	37 (2.8)	502 (3.5)	88 (1.7)	1161 (2.0)
				
*Medications (in previous 13–72 months)*				
Traditional NSAIDs	1771 (25.7)	11 850 (14.6)	6971 (25.8)	47 635 (14.2)
Aspirin	4629 (67.1)	31 068 (38.2)	18 229 (67.6)	122 413 (36.5)
Statins	2290 (33.2)	17 605 (21.7)	9047 (33.5)	70 020 (20.9)
Hormone replacement therapy^b^	324 (24.8)	2965 (20.6)	1102 (21.8)	9871 (17.1)
Oral contraceptive pill^b^	13 (1.0)	510 (3.6)	50 (1.0)	1588 (2.7)
				
*Medications in the last 12 months*				
COX2 inhibitors	1754 (25.4)	1763 (2.2)	7136 (26.5)	5341 (1.6)

Abbreviations: COX2=cyclooxygenase-2; NSAID=non-steroidal anti-inflammatory drug.

a On the basis of cases with colorectal cancer and their controls, only.

b On the basis of female cases with breast cancer and their controls, only.

Values are shown as numbers and %.

**Table 3:** Use of selective COX2 inhibitors (at least one prescription) in cases and in controls in 13 to 72 months before the index date by cancer site

**Cancer**	**Total number of cases**	**Total number of controls**	***No.* of COX2 inhibitors users in cases (%)**	***No.* of COX2 inhibitors users in controls (%)**	**Unadjusted OR (95% CI)^a^**	**Adjusted OR (95% CI)^a^^,^^b^**	**Adjusted *P*-value**
Breast^c^	15 666	62 938	1304 (8.3)	5046 (8.0)	1.09 (1.02–1.17)	1.07 (1.00–1.15)	0.047
Prostate	14 764	61 853	1067 (7.2)	3979 (6.4)	1.16 (1.08–1.24)	1.09 (1.01–1.18)	0.022
Colorectal^d^	11 749	48 624	866 (7.4)	3752 (7.7)	0.97 (0.90–1.05)	0.99 (0.91–1.08)	0.817
Lung	10 163	42 415	845 (8.3)	3500 (8.3)	1.03 (0.95–1.12)	1.00 (0.91–1.09)	0.922
Haematological	7185	29 162	634 (8.8)	2104 (7.2)	1.30 (1.18–1.44)	1.18 (1.07–1.31)	0.001
Bladder	4227	17 559	332 (7.9)	1239 (7.1)	1.17 (1.03–1.34)	1.15 (1.00–1.32)	0.045
Skin	3249	13 115	239 (7.4)	952 (7.3)	1.06 (0.91–1.24)	1.05 (0.89–1.23)	0.579
Oesophagus	3159	13 041	222 (7.0)	941 (7.2)	0.99 (0.85–1.16)	1.03 (0.88–1.21)	0.710
Pancreas	2110	8762	189 (9.0)	716 (8.2)	1.11 (0.94–1.33)	1.12 (0.94–1.35)	0.215
Stomach	1992	8279	143 (7.2)	573 (6.9)	1.07 (0.87–1.30)	1.03 (0.84–1.27)	0.747
All cancers	88 125	362 254	6901 (7.8)	26 974 (7.4)	1.09 (1.06–1.12)	1.06 (1.03–1.09)	<0.001

Abbreviations: CI=confidence interval; COX2=cyclooxygenase-2; OR=odds ratio.

a Compared with no use.

b Adjusted for Townsend quintile, body mass index, smoking status, myocardial infarction, coronary heart disease, diabetes, hypertension, stroke, rheumatoid arthritis, osteoarthritis, use of other lipid-lowering drugs, non-steroidal anti-inflammatory drugs, COX2 inhibitors and aspirin.

c Also adjusted for family history of breast cancer, use of oral contraceptives and hormone replacement therapy.

d Also adjusted for colitis and Crohn's disease.

**Table 4:** Cumulative duration of COX2 inhibitors use in cases and controls in 13–72 months before the index date by cancer site

	**Less than 90 days**		**90 days–12 months**		**13–24 months**		**25 months and more**		
**Cancer**	**Cases/ controls**	**Adjusted odds ratio (95% CI)^a^**	**Cases/ controls**	**Adjusted odds ratio (95% CI)^a^**	**Cases/ controls**	**Adjusted odds ratio (95% CI)^a^**	**Cases/ controls**	**Adjusted odds ratio (95% CI)^a^**	***P*-value^b^**
Breast^c^	684/2888	0.98 (0.89–1.07)	329/1143	1.21 (1.06–1.37)^d^	144/482	1.29 (1.07–1.57)^d^	147/533	1.19 (0.98–1.44)	0.002
Prostate	621/2287	1.11 (1.01–1.22)	225/869	1.04 (0.90–1.21)	109/398	1.11 (0.90–1.38)	112/425	1.09 (0.88–1.36)	0.097
Colorectal^e^	514/2062	1.07 (0.96–1.18)	207/873	1.04 (0.88–1.21)	80/401	0.88 (0.69–1.12)	65/416	0.66 (0.51–0.86)^d^	0.004
Lung	506/1905	1.10 (0.98–1.23)	175/762	0.95 (0.79–1.14)	62/385	0.60 (0.45–0.81)^d^	102/448	0.96 (0.75–1.22)	0.138
Haematological	320/1170	1.09 (0.95–1.25)	169/527	1.25 (1.04–1.50)	72/212	1.31 (0.99–1.74)	73/195	1.47 (1.11–1.95)^d^	<0.001
Bladder	190/662	1.25 (1.05–1.49)	69/297	0.99 (0.75–1.30)	32/139	1.01 (0.68–1.51)	41/141	1.20 (0.84–1.72)	0.369
Skin	137/529	1.06 (0.87–1.30)	54/223	1.00 (0.73–1.36)	32/90	1.49 (0.98–2.27)	16/110	0.68 (0.39–1.16)	0.524
Oesophagus	132/537	1.09 (0.88–1.33)	43/199	0.96 (0.68–1.35)	23/115	0.87 (0.55–1.39)	24/90	1.11 (0.70–1.76)	0.465
Pancreas	97/383	1.07 (0.84–1.36)	53/168	1.37 (0.99–1.90)	18/83	0.95 (0.56–1.61)	21/82	1.08 (0.66–1.78)	0.583
Stomach	86/314	1.13 (0.87–1.46)	33/138	1.01 (0.68–1.51)	14/58	1.01 (0.55–1.84)	10/63	0.67 (0.34–1.32)	0.262
All cancers	3884/15 021	1.07 (1.03–1.11)	1602/6161	1.08 (1.02–1.15)^d^	693/2806	1.03 (0.95–1.12)^d^	722/2986	1.00 (0.92–1.09)	0.236

Abbreviations: CI=confidence interval; COX2=cyclooxygenase-2.

a Adjusted for Townsend quintile, body mass index, smoking status, myocardial infarction, coronary heart disease, diabetes, hypertension, stroke, rheumatoid arthritis, osteoarthritis, use of other lipid-lowering drugs, non-steroidal anti-inflammatory drugs, COX2 inhibitors, aspirin and compared with no use.

b Trend test based on number of months prescribed.

c Also adjusted for family history of breast cancer, use of oral contraceptives, hormone replacement therapy.

d *P*-value<0.01.

e Also adjusted for colitis and Crohn's disease.
