# Supplementary material for: Exposure to cyclooxygenase-2 inhibitors and risk of cancer: nested case–control studies
Source: Br J Cancer. 2011 Jul 12;105(3):452–9. doi: 10.1038/bjc.2011.252 (PMC3172909; doi:10.1038/bjc.2011.252)
Supplement: Supplementary Tables [file bjc2011252x1.doc]

re: Exposure to cyclo-oxygenase-2 inhibitors and risk of cancer: nested case-control studies

Supplement

Table 1 Dose of COX2 inhibitors in cases and controls in 13 to 72 months prior to the index date by cancer site

|  | **Low/Medium** | | | **High** | | |
| --- | --- | --- | --- | --- | --- | --- |
| **cancer** | **Cases /**  **Controls** | **Adjusted**  **Odds ratio (95%CI)** ^#^ | | **Cases /**  **Controls** | **Adjusted**  **Odds ratio (95%CI)** ^#^ | |
| **breast**† | 616/2409 | 1.08 | (0.98 to 1.19) | 688/2637 | 1.08 | (0.99 to 1.18) |
| **prostate** | 493/1895 | 1.06 | (0.95 to 1.18) | 574/2084 | 1.13 | (1.02 to 1.24) |
| **colorectal**‡ | 382/1854 | 0.89 | (0.79 to 1.00) | 484/1898 | 1.10 | (0.99 to 1.23) |
| **lung** | 404/1590 | 1.02 | (0.90 to 1.16) | 441/1910 | 0.97 | (0.86 to 1.10) |
| **haematological** | 303/991 | 1.21 | (1.05 to 1.39) | 331/1113 | 1.17 | (1.02 to 1.33) |
| **bladder** | 156/573 | 1.18 | (0.98 to 1.43) | 176/666 | 1.13 | (0.94 to 1.35) |
| **skin** | 130/492 | 1.10 | (0.89 to 1.36) | 109/460 | 0.99 | (0.79 to 1.24) |
| **oesophagus** | 93/446 | 0.92 | (0.72 to 1.16) | 129/495 | 1.14 | (0.92 to 1.41) |
| **pancreas** | 97/333 | 1.24 | (0.97 to 1.59) | 92/383 | 1.03 | (0.81 to 1.32) |
| **stomach** | 59/268 | 0.91 | (0.67 to 1.22) | 84/305 | 1.16 | (0.90 to 1.51) |
| ***All cancers*** | **3263/12807** | **1.06** | **(1.02 to 1.10)** | **3638/14167** | **1.07** | **(1.03 to 1.11)** |

^#^ Adjusted for Townsend quintile, body mass index, smoking status, myocardial infarction, coronary heart disease, diabetes, hypertension, stroke, rheumatoid arthritis, osteoarthritis, use of other lipid-lowering drugs, NSAIDs, Cox2-inhibitors, aspirin and compared to non-use

† Also adjusted for family history of breast cancer, use of oral contraceptives, hormone-replace therapy

‡ Also adjusted for colitis and Crohn’s disease

§ P-value<0.01

Table 2 Short- and long-term use of COX2 inhibitors by the time of the last prescription in cases and controls in 13 to 72 months prior to the index date by cancer site

|  | **Less than 365 day use**  **Last prescribed before 24 months** | | | **Less than 365 day use**  **Last prescribed in 13 to 24 months** | | | **More than 365 day use**  **Last prescribed before 24 months** | | | **More than 365 day use**  **Last prescribed in 13 to 24 months** | | |
| --- | --- | --- | --- | --- | --- | --- | --- | --- | --- | --- | --- | --- |
| **cancer** | **Cases /**  **Controls** | **Adjusted**  **Odds ratio (95%CI)** ^#^ | | **Cases /**  **Controls** | **Adjusted**  **Odds ratio (95%CI)** ^#^ | | **Cases /**  **Controls** | **Adjusted**  **Odds ratio (95%CI)** ^#^ | | **Cases /**  **Controls** | **Adjusted**  **Odds ratio (95%CI)** ^#^ | |
| **breast**† | 407/1581 | 1.06 | (0.94 to 1.19) | 605/2447 | 1.03 | (0.94 to 1.14) | 222/772 | 1.23 | (1.05 to 1.44) ^§^ | 70/246 | 1.24 | (0.95 to 1.63) |
| **prostate** | 334/1278 | 1.07 | (0.94 to 1.21) | 512/1876 | 1.11 | (1.00 to 1.23) | 155/600 | 1.06 | (0.88 to 1.27) | 66/225 | 1.22 | (0.93 to 1.62) |
| **colorectal**‡ | 290/1176 | 1.06 | (0.92 to 1.21) | 431/1755 | 1.06 | (0.95 to 1.19) | 104/600 | 0.74 | (0.60 to 0.92) ^§^ | 41/221 | 0.81 | (0.58 to 1.14) |
| **lung** | 255/1016 | 1.07 | (0.92 to 1.25) | 426/1650 | 1.05 | (0.92 to 1.18) | 112/614 | 0.74 | (0.59 to 0.92) ^§^ | 52/220 | 0.92 | (0.65 to 1.29) |
| **haematological** | 202/699 | 1.13 | (0.96 to 1.33) | 287/997 | 1.15 | (1.00 to 1.33) | 109/299 | 1.40 | (1.11 to 1.76) ^§^ | 36/109 | 1.33 | (0.90 to 1.96) |
| **bladder** | 99/369 | 1.17 | (0.92 to 1.47) | 160/589 | 1.17 | (0.97 to 1.41) | 56/212 | 1.13 | (0.83 to 1.54) | 17/69 | 1.03 | (0.59 to 1.80) |
| **skin** | 73/305 | 0.98 | (0.75 to 1.29) | 118/446 | 1.09 | (0.87 to 1.36) | 36/150 | 1.06 | (0.72 to 1.56) | 12/51 | 1.03 | (0.54 to 1.95) |
| **oesophagus** | 65/283 | 1.01 | (0.76 to 1.34) | 110/453 | 1.08 | (0.86 to 1.34) | 35/147 | 1.00 | (0.68 to 1.46) | 12/58 | 0.93 | (0.49 to 1.76) |
| **pancreas** | 50/208 | 1.00 | (0.72 to 1.39) | 99/342 | 1.25 | (0.98 to 1.59) | 28/120 | 1.01 | (0.66 to 1.56) | 12/46 | 1.10 | (0.57 to 2.11) |
| **stomach** | 44/190 | 0.99 | (0.70 to 1.39) | 75/262 | 1.17 | (0.89 to 1.55) | 17/82 | 0.88 | (0.51 to 1.51) | 7/39 | 0.73 | (0.32 to 1.68) |
| ***All cancers*** | ***2147/8363*** | ***1.07*** | ***(1.02 to 1.12)*** | ***3337/12804*** | ***1.08*** | ***(1.04 to 1.13)*** ^§^ | ***1027/4291*** | ***0.99*** | ***(0.92 to 1.06)*** | ***390/1516*** | ***1.08*** | ***(0.97 to 1.22)*** |

^#^ Adjusted for Townsend quintile, body mass index, smoking status, myocardial infarction, coronary heart disease, diabetes, hypertension, stroke, rheumatoid arthritis, osteoarthritis, use of other lipid-lowering drugs, NSAIDs, Cox2-inhibitors, aspirin and compared to non-use

† Also adjusted for family history of breast cancer, use of oral contraceptives, hormone-replace therapy

‡ Also adjusted for colitis and Crohn’s disease

* Trend test based on number of months prescribed

§ P-value<0.01

Table 3 Overall use of different types of COX2 inhibitors in cases and controls in 13 to 72 months prior to the index date by cancer site

|  | **Celecoxib** | | |  | **Meloxicam** | | |  | **Rofecoxib** | | |  |
| --- | --- | --- | --- | --- | --- | --- | --- | --- | --- | --- | --- | --- |
| **cancer** | **Cases /**  **Controls** | **Adjusted**  **Odds ratio (95%CI)** ^#^ | | **P-value** | **Cases /**  **Controls** | **Adjusted**  **Odds ratio (95%CI)** ^#^ | | **P-value** | **Cases /**  **Controls** | **Adjusted**  **Odds ratio (95%CI)** ^#^ | | **P-value** |
| **breast**† | 443/1737 | 1.02 | (0.91 to 1.14) | 0.707 | 448/1645 | 1.11 | (0.99 to 1.24) | 0.066 | 550/2099 | 1.07 | (0.97 to 1.19) | 0.184 |
| **prostate** | 334/1264 | 1.05 | (0.92 to 1.20) | 0.448 | 369/1327 | 1.11 | (0.99 to 1.26) | 0.083 | 382/1511 | 0.99 | (0.88 to 1.12) | 0.892 |
| **colorectal**‡ | 318/1246 | 1.13 | (0.99 to 1.29) | 0.065 | 271/1212 | 0.94 | (0.82 to 1.08) | 0.390 | 320/1501 | 0.90 | (0.79 to 1.03) | 0.114 |
| **lung** | 297/1299 | 0.95 | (0.82 to 1.10) | 0.489 | 240/1076 | 0.90 | (0.77 to 1.06) | 0.212 | 342/1369 | 1.04 | (0.90 to 1.19) | 0.627 |
| **haematological** | 227/731 | 1.15 | (0.97 to 1.35) | 0.103 | 221/663 | 1.27 | (1.08 to 1.50) | 0.004 | 250/875 | 1.04 | (0.89 to 1.21) | 0.631 |
| **bladder** | 114/431 | 1.09 | (0.87 to 1.36) | 0.467 | 117/397 | 1.21 | (0.97 to 1.51) | 0.087 | 119/467 | 1.07 | (0.86 to 1.33) | 0.569 |
| **skin** | 81/304 | 1.11 | (0.85 to 1.44) | 0.463 | 79/312 | 1.02 | (0.78 to 1.32) | 0.908 | 97/400 | 1.00 | (0.79 to 1.28) | 0.974 |
| **oesophagus** | 73/315 | 1.02 | (0.78 to 1.34) | 0.878 | 76/314 | 1.03 | (0.79 to 1.35) | 0.813 | 85/349 | 1.05 | (0.82 to 1.36) | 0.688 |
| **pancreas** | 55/242 | 0.90 | (0.66 to 1.23) | 0.504 | 70/247 | 1.20 | (0.90 to 1.60) | 0.208 | 72/285 | 1.05 | (0.79 to 1.40) | 0.712 |
| **stomach** | 48/183 | 1.11 | (0.79 to 1.57) | 0.551 | 35/183 | 0.74 | (0.50 to 1.09) | 0.126 | 64/219 | 1.28 | (0.95 to 1.73) | 0.102 |
| ***All cancers*** | 2318/9169 | 1.03 | (0.98 to 1.08) | 0.258 | 2257/8781 | 1.04 | (0.99 to 1.10) | 0.083 | 2751/10715 | 1.05 | (1.00 to 1.10) | 0.037 |

^#^ Adjusted for Townsend quintile, body mass index, smoking status, myocardial infarction, coronary heart disease, diabetes, hypertension, stroke, rheumatoid arthritis, osteoarthritis, use of other lipid-lowering drugs, NSAIDs, Cox2-inhibitors, aspirin and compared to non-use

† Also adjusted for family history of breast cancer, use of oral contraceptives, hormone-replace therapy

‡ Also adjusted for colitis and Crohn’s disease

* Trend test based on number of months prescribed
